# Editorial for Recent Developments in Emergency Trauma Management

**DOI:** 10.3390/jcm13226683

**Published:** 2024-11-07

**Authors:** Patrick Schober, Georgios F. Giannakopoulos, Lothar A. Schwarte

**Affiliations:** 1 Department of Anesthesiology, Amsterdam University Medical Center, 1081 HV Amsterdam, The Netherlands; p.schober@amsterdamumc.nl; 2 Helicopter Emergency Medical Service‚ Lifeliner 1, 1044 AN Amsterdam, The Netherlands; g.giannakopoulos@amsterdamumc.nl; 3 Department of Surgery, Amsterdam University Medical Center, 1105 AZ Amsterdam, The Netherlands

This Special Issue addresses several specific aspects of emergency trauma management in considerable depth (contributions I–VI). However, before delving into these papers, we would like to provide some over-arching introductory remarks on the topic of emergency trauma management and trauma in general [1]. Furthermore, we will contextualize the papers in relation to each other, and also within the field of emergency trauma management.

Trauma remains a major source of morbidity and mortality worldwide, exerting an enormous burden with respect to medical resources [2,3,4], but also in terms of productivity and individuals’ quality of life. In 2022, the WHO reported that ~1.3 million people die annually due to traumatic injuries sustained in road traffic accidents alone. Moreover, road traffic injuries are estimated to account for 3% of gross domestic product in many countries (WHO, https://www.who.int/news-room/fact-sheets/detail/road-traffic-injuries (accessed on 3 November 2024)). When adding these numbers to all other forms of injury, e.g., falls, burns, shots, or stabbings, the true economic and social burden of trauma becomes only slightly imaginable.

## 1. Diversity

This Special Issue underlines the broad range of topics relevant to emergency trauma management. For example, the papers included in this Special Issue cover both pre-hospital (contribution II) and in-hospital emergency trauma management pathways (contribution IV), reminding us of the chains of assessment and management that are critical in trauma patients. Since every chain is only as strong as its weakest link, it is encouraging to see that current research is aiming to improve emergency trauma management on all fronts.

Furthermore, diversity regarding the trauma population is also covered in this Special Issue. Regarding the age range of the trauma population [5,6], one paper specifically addresses children (contribution I), whereas another focuses on the elderly population (contribution IV). In contrast to most medical conditions, e.g., cardiovascular diseases or cancer, which tend to accumulate with age and gradually become a source of morbidity and mortality, the age-related demographics of trauma are markedly different. For example, there is a high prevalence of head trauma in both the elderly population (e.g., after a fall) and in the younger population, with this trauma often being caused by the aforementioned traffic accidents. With regard to an even younger population of trauma patients [7], we would like to draw attention to the paper by Oude Alink et al., which addresses traumatic spinal injuries in pediatric patients (contribution I). It is evident that, at the other end of the spectrum, elderly trauma patients also present with particular management challenges, including accumulated co-morbidities, relevant medications, or preexisting impairments. Due to the increased aging of many western countries, it is thus increasingly relevant to address the specific features of elderly trauma patients, e.g., frailty. In the context of this Special Issue, we would therefore like to draw attention to the article by Mennen et al., which shares novel insights regarding the management of elderly trauma patients with pelvic fractures (contribution IV). In their contribution, Beijer et al. study the outcome of patients after severe traumatic brain injury by considering both the age and sex of trauma patients [8] (contribution III). Interestingly, the authors found improved outcomes in women aged over 45 years; however, no improvements regarding the patients’ in-hospital or 30-day mortality were demonstrated. We would like to draw attention to this paper, since sex diversity will become a growing topic in trauma care. In general, a more individualized, tailored approach to emergency trauma management that considers the demographic of patients (e.g., age, sex or comorbidities) will become one of the challenges of future trauma care.

As mentioned above, important developments in pre-hospital emergency trauma management are also addressed in this Special Issue. Given that hypovolemia caused by exsanguination is one of the most significant contributors to trauma-related morbidity and mortality, it would be beneficial to establish aside hemorrhage control [3] pre-hospital blood (product) transfusion programs [9]. However, much of the data on this topic derive from non-European or military settings [10] and thus may not be transferable to a civilian context [11]. With this in mind, Jänig et al. (contribution II) studied a large civilian, pre-hospital trauma population to identify patients that could potentially benefit from civil prehospital blood transfusion programs. Certainly, this study is an interesting source for prehospital care providers when discussing prehospital blood (product) transfusion programs [12].

## 2. The Reviews

The two reviews included in this Special Issue span two extremes with regard to the invasiveness of treatments for emergency trauma patients. On the ‘non-invasive’ end of the spectrum, De Grunt et al. systematically review non-invasive analgesia options for trauma patients. This is an important issue, but often not addressed sufficiently in daily praxis [13]. We would like to draw attention to this informative review and promote awareness regarding the necessity of performing adequate analgesia in trauma patients (contribution V).

On the other end of the spectrum, the second review by Schober et al. focusses on traumatic cardiac arrest (contribution VI). Regarding the resuscitation of patients affected by traumatic cardiac arrest, the rapid and aggressive treatment of potentially reversible mechanisms is crucial [14]; these mechanisms often include intravascular hypovolemia due to exsanguination [15]. Invasive resuscitation techniques such as resuscitative thoracostomy are also detailed, all in all providing a concise, current overview of the management of traumatic cardiac arrest.

Both reviews are of interest for a large number of emergency trauma care providers, with analgesia being a routine intervention in many trauma patients and an in-depth knowledge of traumatic cardiac arrest management being a potentially lifesaving skill in these non-routine cardiac arrest cases.

Concluding this Editorial, we would like to thank all authors for their inspiring contributions, all reviewers for the time and effort they have dedicated to improving the preselected manuscripts, and finally the editors and publishers for their continuous support. We hope that we have sparked interest in this Special Issue entitled ‘Recent Developments in Emergency Trauma Management’, and hope that this fine selection of articles inspires its readers.
